# Survival and Germination of *Bacillus clausii* UBBC07 Spores in *in vitro* Human Gastrointestinal Tract Simulation Model and Evaluation of Clausin Production

**DOI:** 10.3389/fmicb.2020.01010

**Published:** 2020-06-10

**Authors:** Jayesh J. Ahire, Megha S. Kashikar, Ratna Sudha Madempudi

**Affiliations:** Centre for Research & Development, Unique Biotech Ltd., Hyderabad, India

**Keywords:** *Bacillus clausii*, UBBC07, spores, SHIME, clausin, GIT

## Abstract

*Bacillus clausii* UBBC07 is a commercial spore probiotic known to reduce diarrhea in children and adults. In the present study, survival and germination of UBBC07 spores were investigated under fed and fasted conditions in Simulator of Human Intestinal Microbial Ecosystem. Besides this, lantibiotic production, purification, and characterization were performed. The agar plate analysis showed that spores were 100% tolerant to fed and fasted gastrointestinal tract (GIT) conditions. Simultaneously, flow cytometry revealed that at the end of small intestinal incubation, 120% (fed) and 133% (fasted) spores were in viable germinating state. The transformation of viable germinating spores into viable vegetative cells was observed at 3 h of incubation under fasted GIT conditions. In antimicrobial evaluation, UBBC07 produced low-molecular-weight (2107.94 Da) class I lantibiotic clausin. The presence of *lanB*, *lanC*, and *lanD* genes confirms the clausin production. Clausin is stable at proteases (pepsin, proteinase K, and trypsin), temperature (up to 100°C), and pH (up to 11). Furthermore, the antimicrobial activity toward Gram-positive bacteria including *Clostridium difficile* is advantageous. In conclusion, *B. clausii* UBBC07 spore probiotic is capable of surviving and germinating under *in vitro* upper GIT conditions. The clausin production justifies strain applicability in diarrhea.

## Introduction

*Bacillus clausii* UBBC07 (MTCC 5472) is a Gram-positive, spore-forming, motile, rod-shaped, non-toxic, and safe probiotic bacterium (Lakshmi et al., 2017) available commercially in the form of spore-suspension and dry formulations in India. Clinically, the consumption of UBBC07 [2 × 10^9^ colony-forming unit (CFU)] capsule (twice a day) for 10 days was found to be safe and effective to alleviate the symptoms of acute diarrhea in adults (Sudha et al., 2013). Similarly, the supplementation of UBBC07 (2 × 10^9^ CFU/5 ml) spore suspension (twice a day) for 5 days effectively reduced the severity of diarrhea in children under 5 years of age (Sudha et al., 2019). No adverse effects and significant changes in safety parameters were reported in UBBC07 clinical investigations (Sudha et al., 2013, 2019). Recently, UBBC07 has been reported to mitigate uremic toxins in the blood of acetaminophen-induced uremia rats (Patel et al., 2019).

Probiotics are the live microorganisms that, when administered in adequate amounts, confer a health benefit on the host (Hill et al., 2014). The success of probiotics depends on the delivery of numbers of active CFU in the gastrointestinal tract (GIT; Ahire, 2012). In this direction, spore probiotics are advantageous as they are capable to survive and germinate under harsh GIT conditions (Casula and Cutting, 2002; Ghelardi et al., 2015). The investigation on spore’s ability to survive and germinate is crucial and a variety of *in vitro* GIT model systems reported their efficiencies as compared to *in vivo* data (Bernardeau et al., 2017; Fois et al., 2019). Simulator of Human Intestinal Microbial Ecosystem (SHIME) is one of the *in vitro* GIT systems (Molly et al., 1993), recognized for quality outcome for more than the last two decades (Van den Abbeele et al., 2010; Elzinga et al., 2019). It has five automated reactors with setup conditions validated according to the *in vivo* parameters of humans and animals (Van den Abbeele et al., 2010). In this study, for the first time, we have investigated the survival and germination of *B. clausii* UBBC07 commercial spore preparation in SHIME.

*Bacillus* spp. are well known for the production of post-translationally modified antimicrobial peptides, i.e., lantibiotics (Willey and van der Donk, 2007). It comprises the characteristic bridges between dehydroalanine (Dha)/dehydrobutyrine (Dhb) and cysteines, referred to as lanthionine (Lan) and methyllanthionine (MeLan; Willey and van der Donk, 2007; Barbosa et al., 2015). To date, gene clusters of class I and II lantibiotics were identified in some strains of *Bacillus* spp. In class I, LanB and LanC enzymes catalyze dehydration and cyclization, whereas in class II, bifunctional synthetase LanM perform both the reactions (Repka et al., 2017). Lantibiotics are active against Gram-positive bacteria including pathogens and antibiotic-resistant bacteria (Field et al., 2015). It has been reported that, lantibiotics bind to bacterial cell wall biosynthesis precursor (lipid II) to arrest cell growth and or form the pores to leak the cellular material (Bouhss et al., 2009). The present study aimed to investigate UBBC07 for the production of novel antimicrobials.

In this study, for the first time, survival and germination of commercial spore probiotic *B. clausii* UBBC07 was investigated under fed and fasted conditions in *in vitro* SHIME model. Lantibiotic (clausin) production, purification, and characterization were reported.

## Materials and Methods

### Survival and Germination of *B. clausii* UBBC07 Spores Under *in vitro* GIT Conditions

Survival and germination of *B. clausii* UBBC07 spore product (2 × 10^9^ CFU/gm) under fed and fasted GIT conditions were investigated by ProDigest model of SHIME (Molly et al., 1993). The model consists of a series of computer-controlled rectors simulating stomach, small intestine, and colon conditions according to the *in vivo* data and modified infogest consensus method (Dressman et al., 1990; Mackie et al., 2016). To evaluate spore germination, an adapted SHIME system representing the physiological conditions of stomach and small intestine within same reactor over time was used. In brief, fed stomach simulation in reactor was achieved by using SHIME media (g/L: starch, 4; yeast extract, 3; mucin, 3; pectin, 2; arabinogalactan, 1.2; peptone, 1; xylan, 0.5; L-cysteine hydrochloride, 0.5; glucose, 0.4) supplemented with pepsin (activity being standardized by measuring absorbance increase at 280 nm of TCA-soluble products upon digestion of reference hemoglobin), phosphatidylcholine (0.17 mM), NaCl, and KCl (levels recommended by consensus method: Mackie and Rigby, 2015). The pH of the reactor was monitored and decreased sigmoidal (from 5.5 ± 0.5 to 2.0 ± 0.5) during the incubation of sample at 37°C for 2 h (stirring). The samples withdrawn at pH 5.5 (0 h) and 2.0 (2 h). During the fasted stomach simulation, the sample was incubated in mucin media containing salts and fourfold lower levels of pepsin and phosphatidylcholine (Riethorst et al., 2016) at 37°C for 45 min (stirring). The samples were removed at 0 and 45 min time interval, where pH (2.0 ± 0.1) remained unchanged (Ahire et al., 2020b).

The fed small intestine simulation had appropriate quantities of bovine bile (10 mM) and pancreatic extracts (pancreatin) ensuring the presence of all relevant enzymes in a specified ratio (SHIME patent conditions, Molly et al., 1993). The sample was incubated at 37°C for 3 h under constant stirring, where pH of the media was immediately increased to 5.5 from 2.0 and later 7.0. At the interval of 1 h (pH 6.5 ± 0.5), 2 h (pH 7.0 ± 0.5), and 3 h (pH 7.0 ± 0.1) incubation, the samples were removed for analysis. The fasted small intestine simulation was achieved as per the recommendations of Riethorst et al. (2016). The sample was incubated at 37°C for 3 h under constant stirring in a media containing five- and three-fold lower levels of pancreatic and bile extracts, respectively. The pH was maintained as described in fed simulation. Samples were withdrawn as described previously.

The samples collected were analyzed for survival and germination by spread plate method and flow cytometry. In brief, samples were diluted in sterile saline (0.85% w/v) and 25 μl of appropriate dilution was spread on tryptone soya agar and incubated at 37°C for 24 h. Simultaneously, the samples diluted in phosphate buffered saline (PBS, pH 7.4) were stained with SYTO 24 and propidium iodide (PI) as per the manufacturer’s instructions and analyzed on BD FACS Verse^TM^ flow cytometer (BD Biosciences, United States). SYTO channel threshold was kept 200 to eliminate signal noise and separate cells from media debris. Parent and daughter gates were appropriately set to determine the population of number of dormant spores (DS), total germinating spores (TGS), viable germinating spores (VGS), non-viable germinating spores (NVGS), total viable vegetative cells (TVVC), and total non-viable vegetative cells (TNVC). Results are reported as average log (counts) ± standard deviation of the three independent biological replicates.

*Bacillus clausii* UBBC07 was tested in triplicate to assess its germination behavior and survival while passing through stomach and small intestine. To each stomach reactor, 2 × 10^9^ spores were added to simulate the ingestion of single dose.

### Screening and Production of Lantibiotic

The 5-μl overnight grown culture of *B. clausii* UBBC07 was spot inoculated on dry surface of Mueller-Hinton (MH) agar (HiMedia, India) and incubated at 37°C for 24 h to develop visible growth. After 24 h, ∼10^5^ cells of *Micrococcus luteus* MTCC 106^*T*^ were seeded in molten MH agar and overlaid on top of visible growth of UBBC07. The plates were incubated at 30°C and observed for zone of clearance.

Besides this, overnight grown single colonies of UBBC07 were each inoculated in 10 ml of MH (pH 7.3 ± 0.1) and clarified-MH broth (pH 7.8 ± 0.1) (Ahire et al., 2020a) and incubated shaking (160 rpm) at 37°C for 24 h. After incubation, the culture was aseptically transferred to 100 ml of respective media and kept under the same conditions as described above. The 10-ml aliquots were removed every after 24 h for up to 196 h. The respective aliquots were centrifuged (Sorvall Legend 65 XTR, Thermo Fisher Scientific, United States) at 11,000 × *g* for 20 min at 4°C and pH of the supernatant was recorded by using DPH 504 pH meter (Global, India). The supernatant was further filter passed [0.2 μm, cellulose acetate (CA) filter, Sartorius, Germany] and investigated for antimicrobial activity against *M. luteus*. In brief, ∼10^5^ cells of *M. luteus* were seeded in molten MH agar and poured into the sterile petri plates (Genaxy Scientific, India). The 25 μl of sample was each dispensed in 9-mm wells created into the agar slab. The plates were kept for radial diffusion at 4°C for 20 min and incubated further at 30°C for 24 h. The zone of inhibition was measured in millimeters.

In another experiment, 24-h-old UBBC 07 cultivated in 10 ml of clarified-MH broth as described above was mixed with 10 g of activated sterile XAD16N (Amberlite, Sigma-Aldrich, United States) beads (van Staden, 2015; Ahire et al., 2020a) and spread evenly on top of sterile clarified-MH agar plate (200 mm × 20 mm, Borosil, India). The plate was incubated at 37°C for 6 days. After incubation, the beads were collected and washed several times with ultrapure water to remove associated growth. Then after, the beads were treated with 30% (v/v) ethanol (CH Fine Chemical, China) and washed several times with ultrapure water. The lantibiotic associated with beads was extracted with 80% isopropanol (IPA; Thermo Fisher Scientific, India) containing 0.1% trifluoroacetic acid (TFA; HiMedia, India) (v/v/v). The extract was filtered through 0.45–and 0.2-μm CA membranes and investigated for antimicrobial activity against *M. luteus* as described above.

### Purification and Characterization of Lantibiotic

The XAD16N beads IPA extract was concentrated using Rotavapor (R-300, Buchi, Switzerland) and purified further by reversed-phase C_18_ cartridge (Sep-Pak, Vac 35 cc, 10 g, Waters, United States) as described by Ahire et al. (2020a). The eluted IPA gradients (10–90% containing 0.1% TFA, v/v/v) were analyzed on thin-layer chromatography (TLC) and antimicrobial activity against *M. luteus*. Biologically active fractions sharing similar TLC profile were concentrated and lyophilized (Lyo lab, United States). The lyophilized samples were stored in umber color bottle at −20°C.

The protein content of lyophilized sample was estimated by Pierce^TM^ bicinchoninic acid (BCA) kit (Thermo Fisher Scientific, United States). UV spectral analysis was performed on Evolution 201 spectrophotometer (Thermo Fisher Scientific, United States). High-performance liquid chromatography (HPLC) was performed by using Agilent LC system (1260 Infinity II, Agilent, United States). In brief, sample (1 mg/ml) prepared in 10% acetonitrile (Thermo Fisher Scientific, India) containing 0.1% TFA (v/v/v) was filtered (0.2 μm CA membrane) and loaded to Premesil^TM^ C_18_ (5 μm, 4.6 mm × 250 mm; Wesley Technologies Inc., United States) column attached to LC. The increasing gradient (B: 25–60%) of acetonitrile containing 0.1% TFA (v/v) was used over 30 min (A: ultrapure water containing 0.1% TFA, v/v) to elute sample at flow rate 1 ml/min. Diode array detector was set to 230 and 254 nm. Peaks collected manually were subjected for antimicrobial activity against *M. luteus*.

Furthermore, the peaks showed antimicrobial activity were subjected for mass analysis using Bruker Daltonics Ultraflex TOF/TOF mass spectrometer (Bruker, United States).

### *In silico* Identification of Lantibiotic Gene Clusters

*Bacillus clausii* UBBC07 whole genome (GenBank accession no. LATY00000000) was analyzed with BAGEL 4 and antiSMASH (Blin et al., 2017), annotated by RAST (Rapid Annotation using Subsystem Technology; Brettin et al., 2015), and visualized with SEED (Overbeek et al., 2014). The NCBI BLAST was performed using default parameters.

### Antimicrobial Activity

Bacterial strains [*M. luteus*, *Listeria monocytogenes* ATCC 49594, *Enterococcus faecium* ATCC BAA-2127, *Streptococcus faecalis* ATCC 29212, *Clostridium difficile* ATCC 9689, *Staphylococcus aureus* MTCC 737, methicillin-resistant *S. aureus* (MRSA) ATCC BAA 1720, *Escherichia coli* MTCC 1687, and *Pseudomonas aeruginosa* MTCC 1688], each grown overnight in their respective growth media, were diluted to ∼10^5^ cells and seeded separately into the molten MH agar to pour the plates. The wells were created, filled with 25 μl of lantibiotic (5 mg dissolved in 1 ml 0.1% TFA water) and incubated as described previously. The zone of growth inhibition was measured in millimeters.

### Determination of Minimum Inhibitory Concentration

The different concentrations (1–256 mg/L) of lyophilized sample were prepared directly in molten MH agar before plating. Suspension of *M. luteus* and MRSA (10^6^ cells/ml) was each spot (2 μl) inoculated onto the dry surface of respective agar plates and incubated as described previously (Andrews, 2001; Ahire et al., 2015). Minimum Inhibitory Concentration (MIC) was defined as the lowest concentration in the agar medium that prevented growth of tested bacteria.

### Stability at Enzymes, Temperature, and pH

One milligram of lyophilized sample was each mixed separately with 1 ml of proteinase K (1 mg/ml; ≥30 U/mg; Sigma-Aldrich, United States) and trypsin (1 mg/ml; 2500 USP/mg; HiMedia, India) prepared in 10 mM Tris–HCl (pH 8.0; Roche, Germany) and incubated at 37°C for 4 h. Similarly, equal quantities of sample and pepsin (1 mg/ml; >3000 NFU/mg, HiMedia, India) were mixed in 0.1% TFA (v/v) water and incubated as described above. After incubation, the mixture was heated at 95°C for 5 min and tested for antimicrobial activity against indicator *M. luteus* (Ahire et al., 2020a).

Aliquots of 0.1% TFA (v/v) water containing 0.1 mg/ml sample were each incubated at 40, 60, 80, and 100°C water bath. At the interval of 10, 30 and 60 min, approximately 1 ml sample was transferred to cool and evaluated antimicrobial activity against *M. luteus* (Ahire et al., 2020a). In another experiment, a 10-ml aliquot was autoclaved at 121°C for 15 min and checked for antimicrobial activity.

Stability at pH was performed by incubating sample (0.1 mg/ml) in ultrapure water having pH 1, 3, 5, 7, 9, 11, and 14 at 37°C for 30 min (Ahire et al., 2020a). After incubation, sample was neutralized to pH 7 and investigated for antimicrobial activity against *M. luteus*.

### Statistical Analysis

All the experiments were performed in triplicate. The Student *t* test was performed by using GraphPad Prism (USA) with a confidence interval of 95%. *P* values of <0.05 were considered statistically significant.

## Results

### Survival and Germination of *B. clausii* UBBC07 Spores Under *in vitro* GIT Conditions

#### Fed Conditions

**FIGURE 1:**
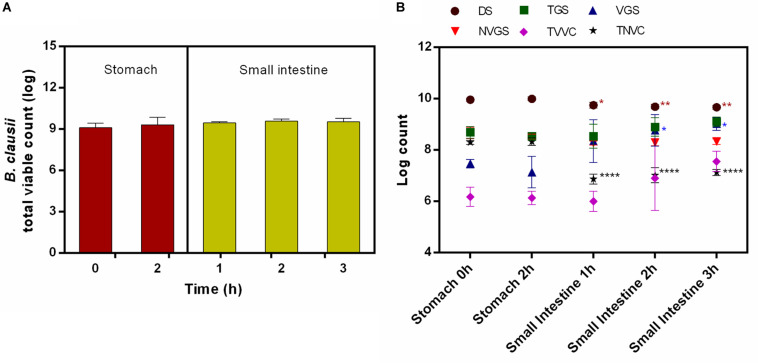
Survival and germination of *B. clausii* UBBC07 in upper GIT under fed conditions. **(A)** Total viable count (TVC) enumerated on agar plate. **(B)** Log count of dormant spores (DS), total germinating spores (TGS), viable germinating spores (VGS), non-viable germinating spores (NVGS), total viable vegetative cells (TVVC), and total non-viable vegetative cells (TNVC) estimated by flow cytometry. Data are expressed as average ± SD of three independent replicates. *p < 0.05, **p < 0.01, and ****p < 0.0001.

*Bacillus clausii* UBBC07 total viable count (TVC) enumerated by agar plate method showed no significant effect of stomach and small intestinal conditions on survival of spores under fed state. No significant changes recorded in TVC during stomach (0 h: 9.10 ± 0.33; 2 h: 9.32 ± 0.54 log) and small intestinal (1 h: 9.46 ± 0.09; 2 h: 9.58 ± 0.15; 3 h: 9.53 ± 0.26 log) incubations (Figure 1A). Simultaneously, the samples analyzed on flow cytometer indicated that the population of DS remained unchanged during stomach simulation (0 h: 9.96 ± 0.06; 2 h: 10.0 ± 0.07 log), whereas it reduced significantly at small intestinal incubation (1 h: 9.75 ± 0.12; 2 h: 9.69 ± 0.09; 3 h: 9.67 ± 0.07 log) (Figure 1B). Besides this, the number of TGS remained unchanged during stomach (0 h: 8.69 ± 0.23; 2 h: 8.54 ± 0.13 log) and small intestinal (1 h: 8.54 ± 0.47; 2 h: 8.90 ± 0.37; 3 h: 9.10 ± 0.19 log) conditions (Figure 1B). The determination of the viable fraction of germinating spores, i.e., VGS, indicated that, the count initially unchanged upon passage through the stomach (0 h: 7.47 ± 0.17; 2 h: 7.14 ± 0.62 log) and increased significantly after the first hour of intestinal incubation (1 h: 8.35 ± 0.83; 2 h: 8.77 ± 0.62; 3 h: 9.02 ± 0.25 log) (Figure 1B). On the contrary, no changes were observed in NVGS during stomach (0 h: 8.67 ± 0.22; 2 h: 8.52 ± 0.10 log) and intestinal (1 h: 8.27 ± 0.08; 2 h: 8.29 ± 0.13; 3 h: 8.33 ± 0.11 log) incubation (Figure 1B). Similarly, TVVC remained unchanged during stomach (0 h: 6.18 ± 0.38; 2 h: 6.13 ± 0.26 log) and intestinal (1 h: 6.0 ± 0.39; 2 h: 6.90 ± 1.29; 3 h: 7.56 ± 0.39 log) incubation (Figure 1B). On the other hand, TNVC count remained unchanged at stomach (0 h: 8.30 ± 0.08; 2 h: 8.33 ± 0.15 log) and decreased significantly under intestinal conditions (1 h: 6.87 ± 0.19; 2 h: 7.02 ± 0.29; 3 h: 7.13 ± 0.12 log) (Figure 1B).

#### Fasted Conditions

**FIGURE 2:**
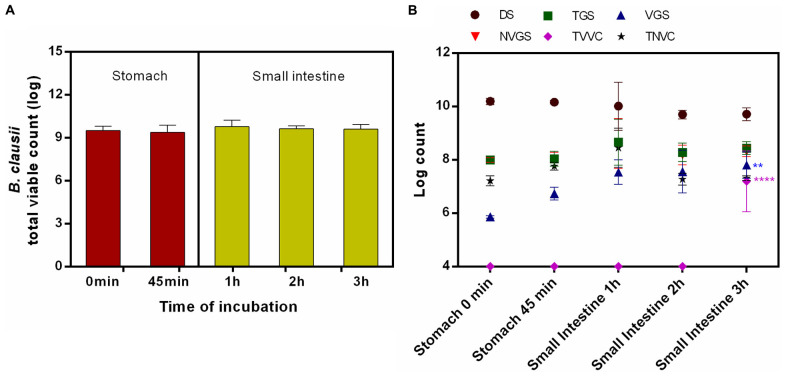
Survival and germination of *B. clausii* UBBC07 in upper GIT under fasted conditions. **(A)** Total viable count (TVC) enumerated on agar plate. **(B)** Log count of dormant spores (DS), total germinating spores (TGS), viable germinating spores (VGS), non-viable germinating spores (NVGS), total viable vegetative cells (TVVC), and total non-viable vegetative cells (TNVC) estimated by flow cytometry. Data are expressed as average ± SD of three independent replicates. **p < 0.01 and ****p < 0.0001.

On agar plate, no statistically significant difference was recorded in survivability of *B. clausii* UBBC07 incubated under fasted stomach (0 min: 9.51 ± 0.30; 45 min: 9.40 ± 0.48 log) and small intestinal (1 h: 9.79 ± 0.45; 2 h: 9.65 ± 0.18; 3 h: 9.61 ± 0.32 log) conditions (Figure 2A). The flow cytometry analysis revealed that, no changes were observed in DS, TGS, VGS, NVGS, and TNVC population during the passage through stomach (0–45 min: DS: 10.19 ± 0.12, 10.16 ± 0.07 log; TGS: 7.99 ± 0.14, 8.04 ± 0.28 log; VGS: 5.86 ± 0.05, 6.73 ± 0.24 log; NVGS: 7.99 ± 0.09, 8.02 ± 0.25 log; TNVC: 7.21 ± 0.18, 7.76 ± 0.15 log) and intestine (1-3 h: DS: 10.01 ± 0.90, 9.70 ± 0.16, 9.71 ± 0.24 log; TGS: 8.66 ± 0.86, 8.28 ± 0.35, 8.44 ± 0.25 log; VGS: 7.54 ± 0.46, 7.56 ± 0.80 log; NVGS: 8.61 ± 0.93, 8.18 ± 0.37, 8.32 ± 0.20 log; TNVC: 8.45 ± 0.73, 7.27 ± 0.22, 7.31 ± 0.09 log) (Figure 2B). The count estimated for VGS (7.80 ± 0.49 log) at 3 h intestinal incubation was significantly higher as compared with stomach. Moreover, log 7.19 ± 1.14 TVVC were detected at the end of small intestinal incubation, which was nil at the beginning of stomach and small intestinal incubation (Figure 2B). This change was statistically significant.

### Screening, Production, and Purification of Lantibiotic

*Bacillus clausii* UBBC07 colony on agar media inhibited the growth of overlaid indicator organism *M. luteus*. However, no activity was observed in supernatants of *B. clausii* cultivated in MH and clarified-MH broth for up to 196 h. During liquid growth, the pH of MH broth was increased from 7.3 ± 0.1 to 7.6 ± 0.01, whereas clarified-MH broth showed a decrease from pH 7.8 ± 0.1 to 7.5 ± 0.05 at 196 h. Bacterial sporulation was observed in both media at 96 h.

The IPA extract of XAD16N beads incubated along with *B. clausii* on clarified-MH agar plate for 6 days exhibited antimicrobial activity (22 ± 0.86 mm) against indicator strain *M. luteus*. The rotavap concentrate of IPA extract showed reduced antimicrobial activity up to 13 ± 0.71 mm. Furthermore, the C_18_ purification yield three active fractions (60, 70, and 80%) which are inhibitory to *M. luteus* (20 ± 1.15, 23 ± 0.57, and 12 ± 0.61) and had equal TLC profile (data not shown).

### Characterization of Lantibiotic

**FIGURE 3:**
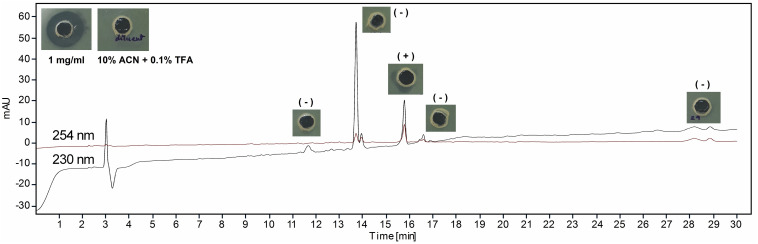
High-performance liquid chromatography (HPLC) analysis of lantibiotic. The insets are the antimicrobial activity of collected peaks, control (10% acetonitrile containing 0.1% trifluoroacetic acid, v/v), and test (1 mg/ml) lantibiotic against *Micrococcus luteus* MTCC 106^*T*^. Data are representative of three independent replicates.

**FIGURE 4:**
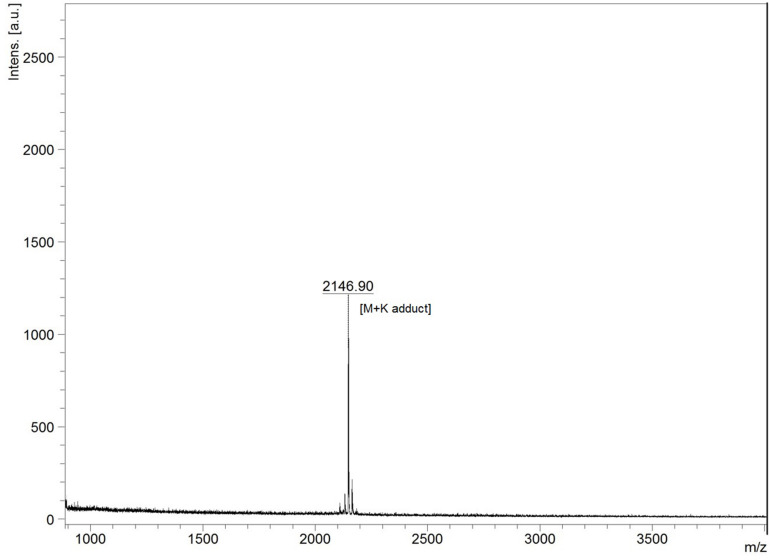
MALDI-TOF mass spectra of HPLC purified lantibiotic of *B. clausii* UBBC07. M + K indicates mass + potassium adduct (2107.94 + 38.96).

The lyophilized sample dissolved separately in TFA (0.1%) water (v/v) and IPA (99%) containing 0.1% TFA (v/v) showed variable reaction with BCA to yield 190.74 ± 26.27 and 487.41 ± 24.52 μg of protein per milligram of sample. In UV spectral scan, the compound showed five major peaks at 219, 222, 230, 235, and 276 nm with absorbance of 1.89, 2.22, 3.01, 2.90, and 1.70, respectively. The HPLC analysis showed that the peak collected at 15.75 min had antimicrobial activity against *M. luteus* (Figure 3). The Ultraflex TOF/TOF-MS revealed that the size of the collected peak was 2146.90 Da, which was detected in the form of M + K adduct (2107.94 + 38.96) (Figure 4).

### Identification of Lantibiotic Gene Clusters in the Genome of *B. clausii* UBBC07

BAGEL4 and antiSMASH failed to indicate the presence of lantibiotic gene clusters. However, RAST visualized and compared in SEED viewer showed the presence of *lanB* and *lanC* gene in *B. clausii* UBBC07, which are 100% identical to the *B. clausii* KSM-K16 strain. The KSM-K16 (AP006627.1) *clausD* (BAD66090.1) amino acid sequence obtained from NCBI blast against UBBC07 in RAST comparative tools showed the 98% identities and 100% positives to hypothetical protein (KKI86113.1; contig LATY01000017), which was located next to the *lanC*.

### Antimicrobial Activity and MIC Determination

**FIGURE 5:**
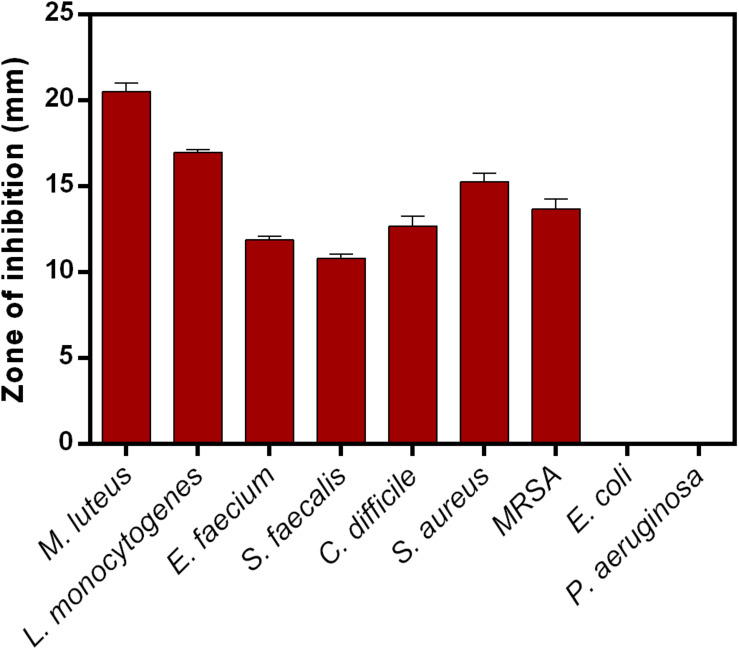
Antimicrobial activity of clausin lanthipeptide (5 mg) against indicator strains. Data points presented are the average of three independent experiments.

Lantibiotic inhibited the growth of Gram-positive bacteria used in this study. The zone of inhibition recorded for *M. luteus*, *L. monocytogenes*, *E. faecium*, *S. faecalis*, *C. difficile*, *S. aureus*, and MRSA are 20.5 ± 0.50, 16.9 ± 0.15, 11.9 ± 0.23, 10.8 ± 0.30, 12.7 ± 0.58, 15.3 ± 0.47, and 13.7 ± 0.58, respectively. No activity was detected against Gram-negative bacteria such as *E. coli* and *P. aeruginosa* (Figure 5). MIC determined against *M. luteus* was 16 mg/L and MRSA 128 mg/L.

### Stability at Enzymes, Temperature, and pH

The proteinase K-, trypsin-, and pepsin-treated lantibiotic showed 100% antimicrobial activity against *M. luteus* as compared to control. In temperature stability studies, lantibiotic was stable up to 100°C with 100% activity. However, activity was completely lost after autoclaving. Besides this, lantibiotic was stable up to pH 11 and showed 100% activity. No activity was detected for pH 14 treated lantibiotic.

## Discussion

Survival and germination are the essential factors for spore probiotics prior to their beneficial effects in GIT. In the present investigation, commercial spore probiotic *B. clausii* UBBC07 enumerated on agar plate showed no significant changes in TVC when incubated under fed and fasting GIT simulations in SHIME, which indicated the ability of spore to survive and germinate under harsh GIT conditions (Casula and Cutting, 2002; Ghelardi et al., 2015; Elshaghabee et al., 2017). However, these results failed to show the real time effects of GIT conditions on spore survival and germination.

The pre-optimized flow cytometry method employed in this study revealed the fate of *B. clausii* spore probiotic under fed and fasted conditions in the SHIME model. At the end of the stomach phase, none of the forms of UBBC07 such as DS, TGS, VGS, NVGS, TVVC, and TNVC were changed significantly from the beginning of stomach incubation, indicating the tolerance of UBBC07 to stomach acid and enzymes. However, no viable cells detected from the start to the end of the stomach phase under fasted state suggested susceptibility of viable vegetative cells to the stomach acid and enzymes in the absence of feed (Ghelardi et al., 2015; Lopetuso et al., 2016).

In a fed state, upon entrance into the small intestine, the number of DS was decreased significantly, indicating that these spores may be transformed into another physiological state. Besides this, the population of VGS increased significantly after an hour of intestinal incubation, indicating the germination of UBBC07 spores upon exposure to the small intestinal conditions. The no change observed with TGS and TVVC suggested favorable conditions of stomach and small intestine in the presence of feed. Comparing the TVC with flow cytometry data for viable cells revealed that the largest fraction of *B. clausii* UBBC07 was present in a viable and culturable state. These results are in agreement that orally ingested *B. clausii* is able to the transit through stomach and germinate in GIT (Lopetuso et al., 2016). Similarly, Ghelardi et al. (2015) have shown *B. clausii* spore germination during *in vivo* GIT transit. Moreover, 120% VGS were estimated at the end of small intestinal incubation under fed condition.

In a fasted state, the number of DS remained unchanged, indicating tolerance, germination, or change in physiological form as observed under fed state. This could be the reason for the significantly increased count of VGS. At the end of small intestinal incubation, a fraction of VGS grew out into TVVC. Around 133% VGS were estimated at the end of small intestinal phase. Therefore, it could be concluded that the largest fraction of the spores, VGSs, and viable bacteria were present in a culturable state. These results are in agreement with a previous finding of *B. clausii* survival, germination, and multiplication as a vegetative form in human gut (Ghelardi et al., 2015; Lopetuso et al., 2016).

The investigation on antimicrobial potential of *B. clausii* UBBC07 showed that the strain is capable of producing lantibiotic only on solid media, which is not in agreement with previous findings on lantibiotic production in both liquid and solid media (Urdaci et al., 2004; Bressollier et al., 2007; van Staden, 2015). It has been reported that lantibiotic production and sporulation coincided and maximum lantibiotic production has occurred when sporulation rate was > 60% (Urdaci et al., 2004). However, in this study, no lantibiotic production was detected at sporulation rate ≥80%. Furthermore, the strain inability to produce lantibiotic in liquid was confirmed by negative activity of IPA extracts (data not shown). Besides this, the production and purification of lantibiotic on clarified-solid media were performed with autoclaved XAD16N beads, which is advantageous, as beads were known to entrap peptide directly from the growing bacteria (van Staden, 2015; Ahire et al., 2020a).

The antimicrobial extracts in IPA were more active as compared to rotavapor aqueous concentrate, suggesting solvent-mediated activity enhancement or solubility (Lee and Kim, 2011). This observation further supported the results of higher protein estimation when dissolved in IPA. The UV spectra indicated the presence of amide and aromatic R-group of phenylalanine in peptide (Field et al., 2007; Aitken and Learmonth, 2009). Furthermore, the molecular weight of clean HPLC peak corresponded well to clausin, a class I lantibiotic known to be produced by *B. clausii* (Urdaci et al., 2004; Bressollier et al., 2007; van Staden, 2015). The *in silico* detection of *lanB* (lanthionine biosynthesis protein), *lanC* (lanthionine biosynthesis cyclase), and *clausD*, i.e., *lanD* (putatively responsible for the oxidative decarboxylation in clausin) (van Staden, 2015), gene in UBBC07 lantibiotic gene cluster and their 100% amino acid identity with *B. clausii* KSM-K16 thus confirmed that the lantibiotic produced by *B. clausii* UBBC07 is indeed clausin.

The clausin lanthipeptide is active against closely related Gram-positive bacteria (Barbosa et al., 2015). It has been reported that, clausin interacts with the lipid intermediate C55-PP-GlcNAc and interferes in the biosynthesis of cell wall polymer teichoic acid (Bouhss et al., 2009). In this study, the ability of clausin to inhibit Gram-positive bacteria is in agreement with previous findings (Urdaci et al., 2004; Barbosa et al., 2015; Field et al., 2015; van Staden, 2015). The antimicrobial activity of UBBC07 against *C. difficile* is advantageous in the treatment of *C. difficile*-associated diarrhea. Besides this, the stability of clausin to proteases (pepsin, proteinase K, and trypsin), temperature (up to 100°C), and pH (up to 11) was maybe due to the presence of unusual amino acids and cyclization (Boto et al., 2018). On the contrary, at the autoclaving temperature (121°C) and pH 14, clausin peptide losing its activity may be due to the disintegration of structure.

## Conclusion

In conclusion, *B. clausii* UBBC07 survived and germinated under fed and fasted GIT conditions in the SHIME model. Approximately, 120% of spores were in viable germinating state during fed and 133% during fasted conditions at the end of small intestinal incubations. Besides this, UBBC07 produced class I lantibiotic clausin with a molecular weight 2107.94 Da. This lanthipeptide is stable at proteases (pepsin, proteinase K, and trypsin), temperature (up to 100°C), and pH (up to 11). Furthermore, the antimicrobial activity toward *C. difficile* is advantageous in the treatment of *C. difficile*-associated diarrhea.

## Data Availability Statement

The raw data supporting the conclusions of this article will be made available by the authors, without undue reservation, to any qualified researcher.

## Author Contributions

JA and RM contributed to the study conception. JA designed the experiments, analyzed the results, and wrote the first draft of the manuscript. JA and MK carried out the experiments. All authors gave intellectual input and critically revised the manuscript.

## Conflict of Interest

JA, MK, and RM are employed by Unique Biotech Limited, India, which is a manufacturer of probiotics. This does not alter our adherence to journal policies on sharing data and materials.
